# Tumor-Promoting Role of GNA14 in Colon Cancer Development

**DOI:** 10.3390/cancers15184572

**Published:** 2023-09-15

**Authors:** Rahui Park, Seungmin Lee, Hyunjung Chin, Anh Thai-Quynh Nguyen, Daekee Lee

**Affiliations:** Department of Life Science, Ewha Womans University, Seoul 03760, Republic of Korea

**Keywords:** CRC, GNA14, G-proteins, β-catenin, MAPK pathway

## Abstract

**Simple Summary:**

In this study, we showed that knockdown of *GNA14* gene, which encodes one of the α subunits of G-protein, inhibits the proliferation of colorectal cancer (CRC) cells harboring truncated *APC* mutations, and that *Gna14* deletion in *Apc^Min/+^* mice attenuates intestinal carcinogenesis through reduced cell proliferation and survival. Knockdown of *GNA14* in CRC cells reduced ERK phosphorylation and β-catenin phosphorylation at S675. Similarly, ERK phosphorylation and β-catenin (S675) phosphorylation in polyps from *Apc^Min/+^* mice were reduced in *Gna14* knockout mice compared to the controls. In sum, this study revealed that GNA14 may promote CRC progression through the ERK and β-catenin pathways.

**Abstract:**

Recent studies have shown that mutations in members of the G-protein α family contribute to the onset and progression of cancer. However, the role of GNA14 in CRC remains unknown. In this study, we examined the effect of GNA14 on CRC through genetic approaches in vitro and in vivo. We found that *GNA14* knockdown by small interfering RNA (siRNA) inhibited the proliferation of CRC cells SW403 and HT29. *Gna14* knockout mice developed normally without obvious abnormalities. However, the number of polyps in the small intestine was significantly reduced in *Gna14* knockout mice compared to control mice after mating with *Apc^Min^* mice, a representative CRC mouse model. In particular, deletion of the *Gna14* inhibited polyp growth, especially in the distal end of the small intestine. Histological examination showed that *Gna14* knockout mice suppressed malignant tumor progression due to decreased proliferation and increased apoptosis in polyps compared to controls. In addition, *GNA14* knockdown in CRC cells resulted in downregulation of ERK phosphorylation and β-catenin and β-catenin phosphorylation at S675. Similarly, ERK phosphorylation and phospho-β-catenin phosphorylation at S675 were decreased in polyps of *Gna14* knockout mice. Collectively, these analyses show that GNA14 may accelerate CRC cell proliferation and malignant tumor progression through ERK and β-catenin pathways.

## 1. Introduction

Heterotrimeric G-protein-coupled receptors (GPCRs) are major cell membrane receptors in eukaryotes, which mediate cellular responses to various external stimuli, including neurotransmitters, hormones, ions, lipids, and light [1]. When activated by specific ligands, GPCRs promote the exchange of GDP to GTP in the Gα subunit of a heterotrimeric G-protein composed of α, β, and γ subunits. G-protein is then dissociated from the GPCRs and subsequently separated into a GTP-bound Gα subunit and a Gβγ subunit, which trigger the activation of various signaling mediators, ultimately transducing cellular signals [2,3]. The Gα subunits are classified into four subfamilies: Gαs, Gαi, Gα12/13, and Gαq. Each subfamily of Gα proteins regulates intracellular effectors, including adenylate cyclase (AC), phospholipase C-β (PLC-β), and RhoGEF, where AC generates cyclic adenosine monophosphate (cAMP) and PLC-β hydrolyzes phosphatidylinositol 4,5-bisphosphate to diacylglycerol (DAG) and inositol 1,4,5-triphosphate (IP3) [4]. Whereas Gαs- and Gαi-induced changes in AC activity transduce signals through the regulation of cAMP-dependent protein kinase A (PKA), the major signaling of Gαq is mediated through the products of PLC-β, DAG, and IP3, which activate protein kinase C (PKC) and calcium ion transport, respectively [4]. Meanwhile, the dissociated Gβγ subunit can also regulate AC and PLC-β, which are known as major downstream effectors of the Gα subunit; thus, while G-protein downstream signaling is not specific to the Gα subfamily, redundant signaling events can occur simultaneously depending on the cellular context [5].

As research has gradually revealed the functions of GPCRs for cancer cell growth and metastasis, the likelihood of developing anticancer drugs targeting GPCRs and G-protein has increased [6,7]. Recent investigations have highlighted the role of Gαq subunits encoded by *GNAQ, GNA11, GNA14,* and *GNA15* in cancer development. Mutations in *GNAQ* and *GNA11* are best known in uveal melanoma, and about 80–90% of uveal melanomas have missense mutations that maintain either Gαq or Gα11 in an active state [8]. Moreover, activating mutations in *GNA14* have been found in various vascular tumors [9,10,11] and hepatic small vessel neoplasm [12], and research has revealed higher expression of GNA14 in endometrial carcinoma tissues than in hyperplasia tissues [13]. Activation of GNA14 by ligand treatment or substitution mutations in *GNA14* activates the MAPK pathway, an important signaling pathway for cell proliferation, in HepG2, neonatal human melanocytes, and human umbilical vein endothelial cells, respectively [9,14]. On the other hand, studies have found that decreased expression of *GNA14* due to DNA hypermethylation are closely related to the progression of hepatocarcinoma [15,16]. Finally, *GNA15* has been shown to be significantly overexpressed in gastrointestinal neuroendocrine tumors and pancreatic ductal adenocarcinoma [17,18].

CRCs remain a common malignancy in humans worldwide, and the mortality and incidence of CRC has increased over the past years [19]. Research has established that aberrant overexpression or mutation of limited GPRCs is related to tumor growth and metastasis [4,7]. Although various GPCRs are expressed in colorectal cancer cells, the roles of GPCRs and G-proteins as cancer drivers involved in various cellular signaling including the MAPK pathway have recently been identified in CRC cells [7]. In addition, previous research has reported that 2.7% of colorectal cancers have somatic mutations in GNA14 [20]. Analysis of GEO datasets using GEO2R has shown that while *Gna14* is one of the genes whose expression increases during progression from adenoma to carcinoma in *Apc^Min^* mice [21], the role of GNA14 in colorectal cancer remains unknown. In this study, we analyzed the biological function of *GNA14* in colon cancer using the *GNA14* knockdown method in CRC cells and by crossing *Gna14* knockout mice with *Apc^Min^* mice, a representative mouse model of CRC.

## 2. Materials and Methods

### 2.1. Cell Lines and Culture

Human colorectal cancer cells including HCT-15, DLD-1, LoVo, SW403, and SW620 cell lines were purchased from Korean Cell Line Bank (Seoul, Republic of Korea). HT-29, HCT116, RKO, SW48, and SW480 cell lines were purchased from American Type Culture Collection (Manassas, VA, USA). HCT-15, DLD-1, LoVo, SW403, SW620, SW48, and SW480 cells were maintained in RPMI-1640 medium, HT-29 and HCT116 cells were grown in McCoy’s 5A medium, and RKO cells were cultured in Dulbecco’s modified Eagle’s medium. Media was supplemented with 10% fetal bovine serum (FBS), and cells were maintained at 37℃ in a humidified atmosphere of 5% CO_2_.

### 2.2. Transient Transfection of siRNA

Cells were seeded into 6-well plates before 18 h transfection. Two small interfering RNAs (siRNAs) designed to target *GNA14* were transiently transfected using Lipofectamine RNAiMAX reagent (ThermoFisher Scientific, Waltham, MA, USA) according to the manufacturer’s instruction. The *GNA14* knockdown siRNA, siGNA14-1 (5′-CAGCUAAACCUAAGGGAAU-3′) with 3′-UU overhangs, was synthesized by Genolution (Seoul, Republic of Korea) and siGNA14-2 (5′-CGAUGGACACGCUAAGGAUACAGTA-3′) with 2-base 3′-overhangs was synthesized by Integrated DNA Technologies (IDT, Coralville, IA, USA), respectively. Universal negative control DsiRNA (IDT, 51-01-14-03) was used as negative control siRNA. Each siRNA or an equal amount of the siRNA mixture (siGNA14-1 and siGNA14-2) was treated in the cells to a final concentration of 20 nM.

### 2.3. Cell Proliferation Assay

To quantify the cell proliferation, the live cells were counted after staining with trypan blue solution (Sigma-Aldrich, St. Louis, MO, USA).

### 2.4. Wound Healing Assay

Cells transfected with *GNA14* siRNA for 24 h were completely resuspended in medium and cultured for 24 h in SPLScar™ blocks with 0.5 mm cell-free gaps (SPL Life Sciences 201935, Seoul, Republic of Korea). After removing the block, the cells were gently washed twice with PBS and supplemented with medium. The same area was photographed immediately after block removal and 24 h later. The ImageJ program was used to measure and quantify three different wound healing areas.

### 2.5. RNA Extraction and Quantitative Reverse Transcription-Polymerase Chain Reaction (qRT-qPCR)

Total RNA was isolated from cells or tissues using TRIzol^®^ reagent (ThermoFisher Scientific) according to the manufacturer’s protocol. Total RNA (1 µg) mixed with random hexamer (2.5 µM) was synthesized into cDNA using Superscript™ IV Reverse Transcriptase (ThermoFisher Scientific). RT-qPCR was performed with an ABI 7900HT Fast Real-Time PCR System (ThermoFisher Scientific) using a KAPA SYBR FAST qPCR Master Mix kit (Kapa Biosystems, Wilmington, MA, USA) according to the manufacturer’s instructions. Real-Time PCR primer sequences are listed in the Supplementary Materials (Table S1). Relative steady-state levels of the targets were quantified using the 2^–∆∆Ct^ method.

### 2.6. Western Blot Analysis

Proteins were extracted from human cells and mouse tissues using RIPA buffer. Protein quantification, SDS-PAGE, and Western blot were performed as previously described [22,23]. Sources and dilutions of the antibodies used in this study are listed in the Supplementary Materials (Table S2). Protein band density was quantified using the ImageJ program.

### 2.7. Construction of Gna14 Knockout Mice Using CRISPR-Cas9 System and Crossing of Mice

*Gna14* sgRNA was synthesized with the MEGAshortscript™ T7 kit (ThermoFisher, AM1354) according to the manufacturer’s protocol. The target sequences of *Gna14* sgRNA are 5′-ACGTGGTATTTCCTTTCCGT-3′ and 5′-CCAAATAGTGAGTACTCAGC-3′ were located in introns 2 and 3, respectively. As previously described [24], a mixture of sgRNA (20 ng/μL of each) and SpCas9 nuclease V3 (50 ng/μL, IDT, Coralville, Iowa) in Opti-MEM (ThemoFisher) was electroporated into fertilized one-cell embryos. One-cell embryos were obtained from superovulated C57BL/6N (B6) females crossed with B6 males. Electroporated eggs were implanted into the oviduct of pseudopregnant ICR females. Initial screening of deletion mutants was analyzed by agarose gel electrophoresis after PCR using the DNA extract of the founder mouse. The primer sequences for PCR genotyping are 5′-CCAAATCACCCCGCCTAGT-3′ (sense) and 5′-TCTCTGCCTCCTGGGAAGT-3′ (antisense). The deleted portion was identified by performing TA cloning and sequencing as previously described [25]. *Gna14^+/−^* mice were crossed with *Apc^Min/+^* mice (Stock No:002020; Jackson Laboratory, Bar Harbor) to generate *Gna14^+/−^, Apc^Min/+^* mice. These mice were then further crossed with *Gna14^+/−^* to obtain *Gna14^+/+^* and *Gna14^−/−^* in *Apc^Min/+^* background, respectively. All mice were maintained under specific pathogen-free conditions, and all mouse experiments were approved by the Institutional Animal Care and Use Committees (IACUC) of Ewha Womans University.

### 2.8. Polyps Counting and Histological Evaluation

For microscopic evaluation of polyps, mice were sacrificed using CO_2_ asphyxiation at 5 months of age, and their small intestines and colons were collected and processed as previously described [23]. Each small intestine was divided into three equal parts in length (proximal, middle, distal) for ease of handling. The detailed procedure for histological evaluation of Swiss-rolled ileum has been previously described [23].

### 2.9. BrdU, TUNEL Staining, and Immunohistochemistry

To observe cell proliferation in the intestine, 5-bromo-2′-deoxyuridine (BrdU, Sigma-Aldrich) were injected intraperitoneally (0.1 mL of 10 mM BrdU in PBS per 10 g body weight) 2 h before sacrifice. For BrdU immunostaining, rehydrated paraffin sections (5 µm) were boiled for 10 min in 10 mM sodium citrate buffer, pH 6.0, for antigen retrieval. Tissue sections were then treated with 3% H_2_O_2_ in deionized water for 10 min and immunostained using Vectastatin Elite ABC kit (Vector Lab, PK-6100) according to the manufacturer’s protocol. Anti-BrdU antibody (1:100) was purchased form Abcam (ab6326, Cambridge, UK). Apoptotic cells were detected using TUNEL staining kit (Merck, S7100) according to manufacturer’s protocol. Finally, antigen–antibody complexes were visualized using the DAB+ substrate chromogen system (Dako, K3467). Immunohistochemistry was performed in a manner similar to the BrdU staining described above. Briefly, endogenous peroxidase quenched slides were incubated with blocking buffer containing 5% normal goat serum in TBST (20 mM Tris-HCl, pH 7.5, 150 mM NaCl, 0.05% Tween-20) for 1 h at room temperature. Slides were then treated with anti-phospho-ERK1/2 (1:1000; CST, #9101) or anti-phospho-β-catenin (1:500; S675, CST, #4176) antibody in blocking buffer overnight at 4 °C. After successive washes in TBST and secondary antibody treatment, signals were detected as described above. The relative degree of immunostaining in the polyps was quantitatively analyzed using the ImageJ program.

### 2.10. Statistics Analysis

Statistical analysis was performed using GraphPad Prism 7 software. All experiments are represented as mean ± SEM. To analyze the difference, unpaired two-tailed Student’s *t*-tests or one-way ANOVA were performed. *p*-values *, <0.05; **, <0.01; and ***, <0.001 were considered significant.

## 3. Results

### 3.1. GNA14 Knockdown Inhibited the Proliferation of Colorectal Cancer (CRC) Cells, but Did Not Affect Cell Migration

**Figure 1:**
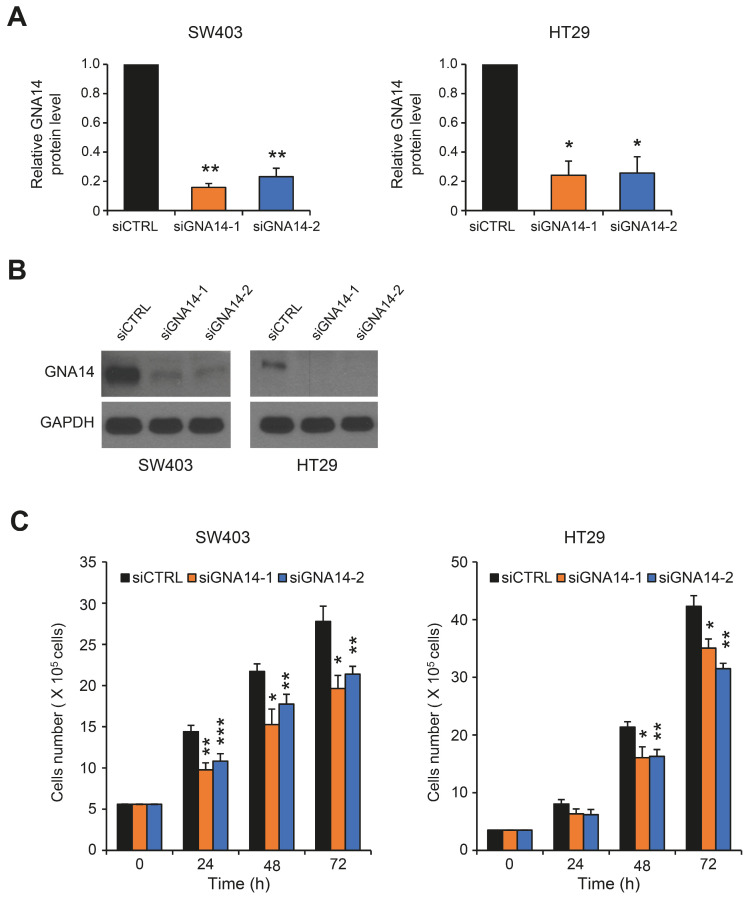
Effect of *GNA14* knockdown on cell proliferation in colon cancer cells. (**A**) Cells (SW403 and HT29) were transfected with siRNAs for 48 h, and GNA14 protein levels were analyzed using Western blotting (*n* = 2). (**B**) Images are representative Western blots after siRNA treatment. (**C**) Cells were stained with trypan blue 24, 48, and 72 h after transfection with siRNA, and viable cells were counted. Each bar represents mean ± SEM (*n* = 4). The two-tails unpaired Student’s *t*-test was used. * *p* < 0.05, ** *p* < 0.01, *** *p* < 0.001. Original western blots are presented in Supplementary Materials File S1.

Previous study has shown that silencing of GNA14 inhibits endometrial cancer cells’ proliferation by inducing apoptosis and G_2_/M cell cycle arrest [13]. To investigate the role of GNA14 in colorectal cancer, we first examined *GNA14* gene expression in several CRC cells (Figure S1A,B). SW403 and HT29 cells showed relatively high expression compared to other cells. These two cells do not show any unique molecular features that make them different from other colon cancer cell lines with respect to GNA14 expression, but they do share the commonality of being colon-like cell lines [26]. SW403 and HT29 cells were then transiently transfected with control siRNA (siCTRL) or siRNA targeting *GNA14* (siGNA14-1, siGNA14-2). Both *GNA14* siRNAs efficiently reduced GNA14 protein levels (Figure 1A,B). Cell proliferation was analyzed using trypan blue staining, and cell proliferation was reduced in both SW403 and HT29 cell lines transfected with *GNA14* siRNA compared to control siRNA (Figure 1C). Metastasis is known to be a leading cause of death in colorectal cancer patients. To analyze whether migration of SW403 and HT29 cells is dependent on *GNA14* expression, we performed the wound healing assay. Knockdown of *GNA14* did not significantly affect the migration abilities of either cell (Figure S2A–D).

### 3.2. Gna14 Knockout Mice Developed Normally

**Figure 2:**
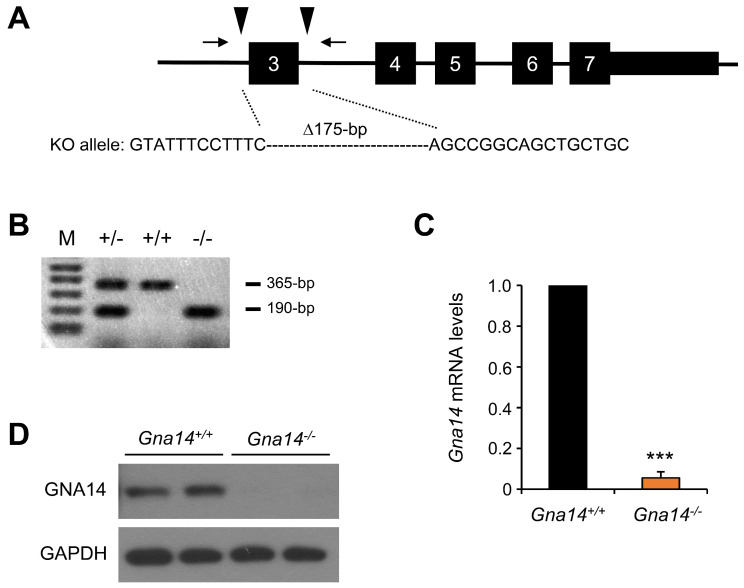
Generation of *Gna14* knockout mice. (**A**) The mouse *Gna14* gene structure is shown. Numbers indicate exons, arrowheads indicate sgRNA targets, and arrows indicate PCR genotyping primers, respectively. The *Gna14* KO allele had a 175 bp deletion including the entire exon 3. (**B**) PCR products from genomic DNA of wild-type (+/+), heterozygote *Gna14^+/−^* (*+/−*), and *Gna14^−/−^* (−/−) mice were analyzed using agarose gel electrophoresis (wild-type allele, 365 bp product; *Gna14* KO allele, 190 bp product). M, 1 kb plus DNA ladder. (**C**) Real-time PCR analysis was used to measure *Gna14* mRNA levels in the kidney (*n* = 3), *** *p* < 0.001 (two-tails unpaired Student’s *t*-test). (**D**) Kidney tissue extracts were analyzed with Western blot using indicated antibodies (each lane represents tissue from a different mouse). Original western blots are presented in Supplementary Materials File S1.

To investigate the in vivo function of GNA14, we constructed a *Gna14* knockout mouse by deleting exon 3 using the CRISPR/Cas9 system (Figure 2A). Founder mice carrying the deleted *Gna14* allele were crossed with wild-type mice to generate heterozygous mice carrying a mutant allele with a 175 bp deletion including the entire exon 3. As a result of PCR genotyping using the DNA lysate of pups obtained from the crossing of heterozygous *Gna14* mice, the wild-type showed a 365 bp PCR product and the *Gna14* mutant allele showed a 190 bp PCR product, so the genotype was further determined using PCR (Figure 2B). Relatively, *Gna14* mRNA (Figure 2C) was at basal levels in the kidney of knockout mice, and GNA14 protein was no longer expressed in the kidney of knockout mice (Figure 2D). *Gna14* knockout mice developed normally and their reproduction was normal in adulthood.

### 3.3. The Number of Small Intestine Polyps Was Reduced in Gna14 Knockout Mice

**Figure 3:**
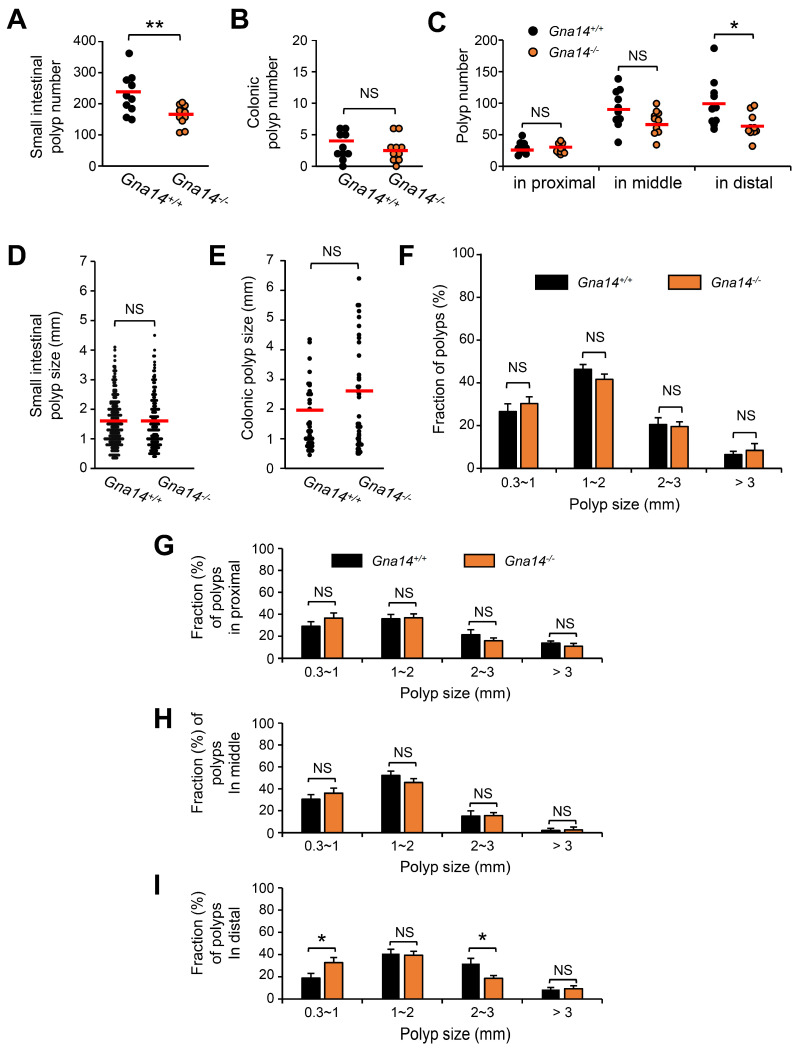
Effect of *Gna14* on *Apc^Min^* intestinal tumorigenesis. The number of small intestinal polyps (**A**) and colonic polyps (**B**) were counted in *Apc^Min^* mice of 5-month-old wild-type or *Gna14* knockout background. Each dot represents the total number of polyps in wild-type (*n* = 10) and *Gna14* knockout mice (*n* = 10). The horizontal lines represent the mean numbers of polyps. (**C**) Distribution of polyp number according to small intestine location. After obtaining the average value for each polyp size (in mm) in all mice (*n* = 10 per genotype), the polyp size was displayed in a graph by changing it to an integer point (**D**) in the small intestine and (**E**) in the colon. (**F**) Relative percentages corresponding to a given polyp size range were calculated in each mouse, and the average value according to genotype was expressed as means ± SEM. (**G**–**I**) The relative distribution ratios according to polyp size are shown by dividing the small intestine into proximal, middle, and distal part. The two-tails unpaired Student’s *t*-test or one-way ANOVA with Tukey’s multiple comparisons test were used. NS means statistically nonsignificant. * *p* < 0.05, ** *p* < 0.01.

To investigate the role of *Gna14* in intestinal tumorigenesis, *Gna14^+/−^* mice were crossed with *Apc^Min/+^* mice. Thereafter, the number and size of polyps generated in the intestine of 5-month-old mice were measured under a dissecting microscope. Interestingly, the number of polyps was significantly reduced in *Gna14* knockout mice compared to wild type in the small intestine, but no difference was found in the colon (Figure 3A,B). The number of polyps in the small intestine of *Apc^Min/+^* mice is known to have a non-uniform distribution, resulting in more polyps occurring at the distal end [27,28]. In the control mice, more polyps occurred in the distal part of the small intestine, and a similar number of polyps were observed in the proximal and middle parts of the small intestine regardless of the *Gna14* genotype (Figure 3C). However, at the distal end, the number of polyps was significantly reduced in *Gna14* knockout mice compared to the control mice (Figure 3C). On the other hand, the sizes of the tumors in the small intestines and colons of control and *Gna14* knockout mice were not significantly different (Figure 3D,E). Moreover, the proportion of polyps depending on size in the small intestine of control and *Gna14* knockout mice were not significantly different (Figure 3F). Similarly, regardless of the *Gna14* genotype, the relative distributions according to polyp size were similar in the proximal and middle parts of the small intestine (Figure 3G,H). However, the proportion of polyps less than 1 mm in diameter at the distal part was significantly higher in *Gna14* knockout mice than in control mice (18.8 ± 3.6% and 32.7 ± 3.5%, respectively) (Figure 3I). Conversely, the proportion of tumors between 2 mm and 3 mm in diameter was smaller in *Gna14* mice than in control mice (31.8 ± 4.9% and 18.6 ± 2.9%, respectively), indicating that GNA14 contributes to polyp growth from the early stages in the distal part of the small intestine.

### 3.4. Tumor Progression was Attenuated in Gna14 Knockout Mice

**Figure 4:**
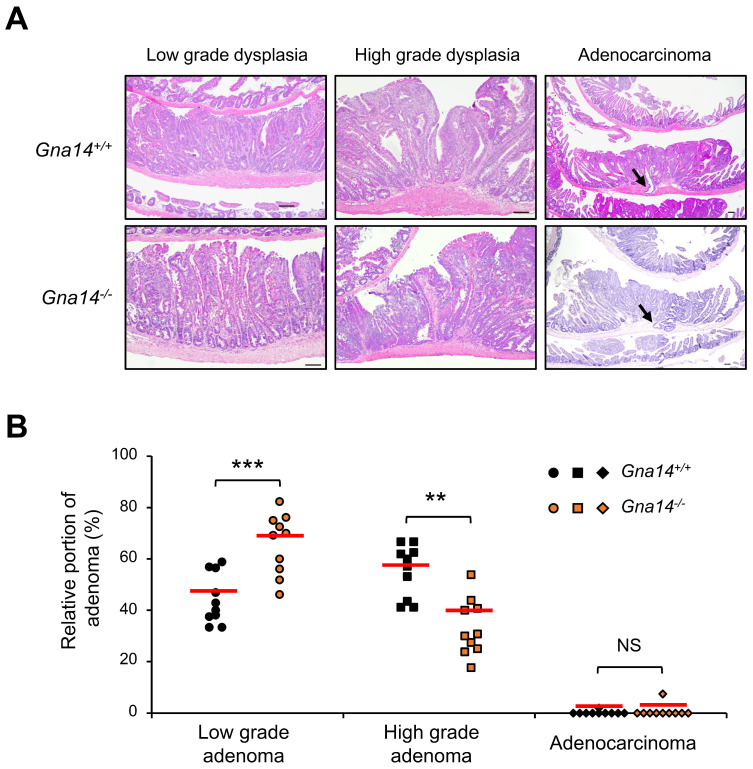
Histological diagnosis in distal small intestine polyps. (**A**) Representative H&E staining of adenomas according to histological grade is shown. Arrows indicate submucosal invasion. Scale bar, 100 µm. (**B**) Each dot represents relative percentages of low- and high-grade adenoma and adenocarcinoma in wild-type and *Gna14* knockout mice, respectively. The horizontal lines represent the means of the percentages of adenoma (*n* = 10 per genotype). The one-way ANOVA with Tukey’s multiple comparisons test was used. NS means statistically nonsignificant. ** *p* < 0.01, *** *p* < 0.001.

**Figure 5:**
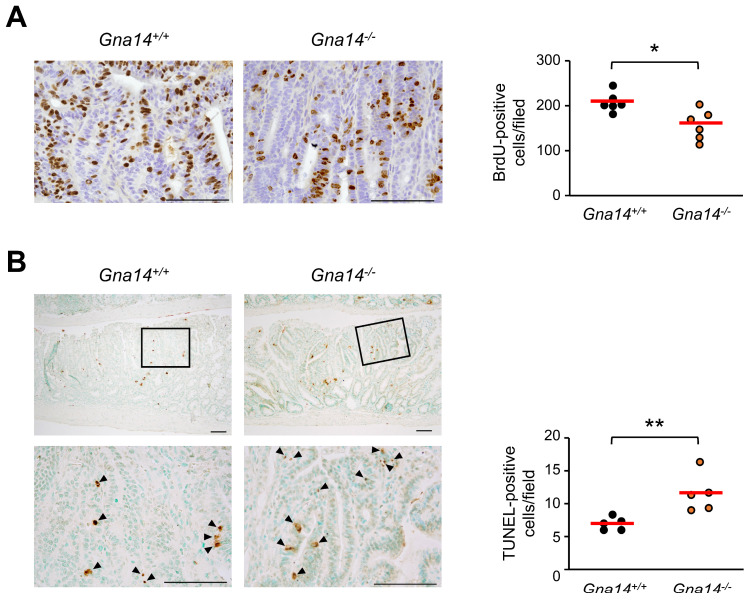
Effect of *Gna14* on cell proliferation and apoptosis in polyps. (**A**) Representative images of BrdU-stained intestinal polyp are shown. Numbers of BrdU-positive cells were counted in intestinal polyps from wild-type (*n* = 6) and *Gna14* knockout mice (*n* = 6). Each dot (right) is the average number of BrdU-positive cells in each mouse. The horizontal lines represent the averages for all mice. (**B**) Representative images of TUNEL-stained intestinal polyp are shown. The pictures below are enlarged pictures of the rectangular part. Numbers of TUNEL-positive cells (arrowheads) were counted in intestinal polyps (*n* = 5 per genotype). Each dot (right) is the average number of TUNEL-positive cells in each mouse. The horizontal lines represent the averages for all mice. * *p* < 0.05, ** *p* < 0.01, two-tails unpaired Student’s *t*-test. Scale bar = 50 μm.

Because the number and size of polyps between control and *Gna14* knockout mice differed significantly in the distal small intestine, polyp grade was assessed using histopathological analysis of the distal small intestine. Adenoma grades in control and *Gna14* knockout mice were classified into low-grade adenoma, high-grade adenoma, and adenocarcinoma. Figure 4A shows representative H&E staining images. Compared to the control mice, *Gna14* knockout mice had a higher proportion of low-grade adenoma and a lower proportion of high-grade adenoma. However, the analysis revealed no significant difference in the rate of adenocarcinoma between the two groups (Figure 4B). To identify the cause of the difference in polyp growth, cell division and apoptosis were investigated using BrdU staining and TUNEL analysis, respectively. Compared to the control mice, *Gna14* knockout mice had a lower ratio of BrdU staining polyps (Figure 5A) and significantly higher apoptosis (Figure 5B), suggesting that GNA14 contributes to cell proliferation and survival during intestinal carcinogenesis. However, the analysis revealed no difference in cell division between the two groups in normal crypt (Figure S3A,B).

### 3.5. Decreased Phosphorylation of ERK and CTNNB1 upon GNA14 Reduction

**Figure 6:**
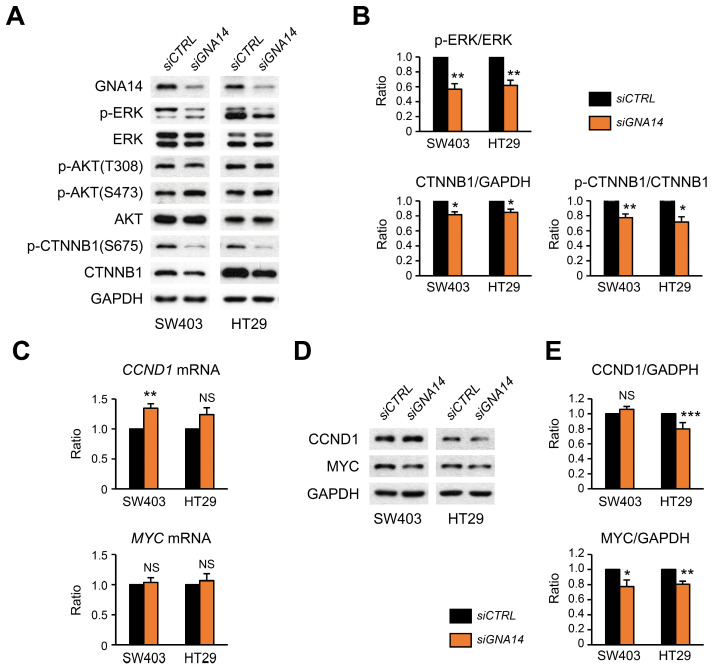
Effect of *Gna14* knockdown on downstream signaling pathways in colon cancer cells. Cells (SW403 and HT29) were transfected with 20 nM of control siRNA (*siCTRL*) or a mixture of *GNA14* siRNA (*siGNA14*) for 48 h. (**A**) Changes in signaling pathways leading to cell proliferation were analyzed using Western blotting. Representative blots are shown here. (**B**) The difference in signal intensity of Western blots by siCTRL and siGNA14 was quantified using the ImageJ program and is shown as a bar graph (*n* = 3). (**C**) Relative static levels of mRNA are shown (*n* = 4). (**D**) Representative Western blots are shown, and (**E**) the difference in signal intensity of Western blots by siCTRL and siGNA14 was quantified (*n* = 4). Each bar represents mean ± SEM. The two-tails unpaired Student’s *t*-test was used. NS means statistically nonsignificant and * *p* < 0.05, ** *p* < 0.01, *** *p* < 0.001. Original western blots are presented in Supplementary Materials File S1.

**Figure 7:**
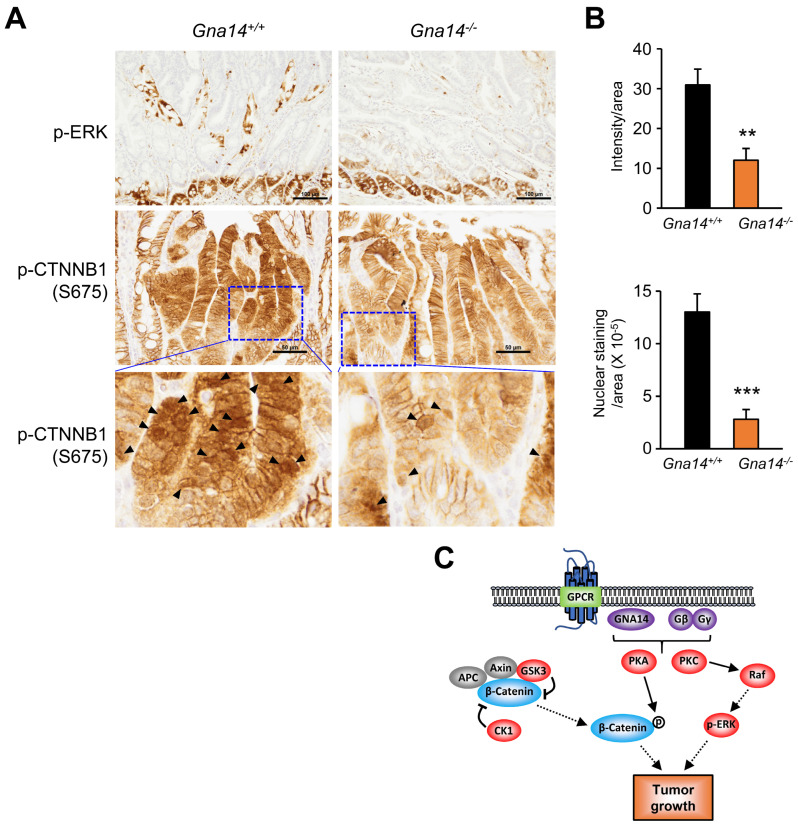
Effect of *Gna14* knockdown on phosphorylation of ERK and β-catenin in the polyp. (**A**) Immunohistochemistry of phospho-ERK (200×) and phospho-β-catenin at S675 (400×) in the distal region of polyps. Arrowheads indicate representative nuclear staining of phospho-β-catenin. The blue squares are enlarged at the bottom. (**B**) Quantification of immunohistochemistry of phospho-ERK (200×) and phospho-β-catenin at S675 (400×) shown in A. In each polyp site, the extent of immunostaining for phospho-ERK (*n* = 6~7 per genotype) and the number of intranuclear immunostaining for phospho-β-catenin at S675 (*n* = 6 per genotype) in a given area were quantified using the ImageJ program. Each bar represents mean ± SEM. Two-tails unpaired Student’s *t*-test was used. ** *p* < 0.01, *** *p* < 0.001. (**C**) A schematic diagram of the effect of GNA14 on colorectal cancer.

To determine the cause of decreased cell proliferation due to *GNA14* knockdown, we investigated potential changes in the MAPK and AKT pathways, two of the most important signaling pathways involved in the proliferation and survival of colorectal cancer cells. In both HT29 and SW403 cells, ERK phosphorylation was decreased due to *GNA14* knockdown, but no change was found in AKT phosphorylation at T308, downstream of PI3K pathway. Interestingly, both the protein amount of β-catenin and phosphorylation of β-catenin at S675 were reduced due to *GNA14* knockdown (Figure 6A,B). Contrary to expectations, the static levels of mRNA for CCND1 and MYC, two of β-catenin’s classic target genes, did not decrease (Figure 6C), but the amount of MYC protein decreased significantly in both cell lines, and for CCND1 protein, only in HT29 cells (Figure 6D,E), suggesting a regulatory mechanism at the translational level. Meanwhile, both immunostaining of phospho-ERK in adenomas of the distal small intestine and immunostaining of phospho-β-catenin at S675 in the nucleus were reduced in *Gna14* knockout mice compared to controls (Figure 7A,B).

## 4. Discussion

GPCRs are the largest superfamily of molecules on a cell surface, accounting for about 2% of all genes encoded in humans, and activation of GPCRs by diverse ligands drives numerous cellular and physiological processes in normal tissues. Although GPCRs play a relatively unremarkable role in cancer development compared to growth factor receptor signaling abnormalities, researchers have increasingly elucidated their role as cancer drivers, with dysregulation of GPCRs in various cancer types leading to malignant cell growth and angiogenesis [2,6,7]. Meanwhile, studies of GPCR signals over the past few decades have shown that, rather than simple binary switches, they are allosteric microprocessors that deliver biased signals [29,30]. In addition, the combination of different G proteins among the various subunits of the G protein subfamily contributes to the diversity of downstream signaling pathway. Recent studies have actively pursued the development of anticancer drugs targeting GPCRs and G proteins [4,6,31].

The main downstream signaling of Gαq in cancer is likely mediated through PKC, which activates numerous enzymes and transcription factors involved in carcinogenesis [32]. However, the downstream cell signaling mechanisms by which GNA14 affects cancer cell proliferation are less well understood. Previous research has shown that the mitogenic signaling pathway of bradykinin in the human colon cancer cell line SW480 is mediated by the sequential activation of Gαq, PI3K, and PKC [33]. In the present study, we found reduced high-grade dysplasia in *Gna14* knockout polyps and reduced MAPK phosphorylation upon *GNA14* knockdown in human colon cancer cell lines or in polyps from knockout mice, suggesting that GNA14-induced MAPK phosphorylation may be associated with colon cancer cell proliferation. The most reasonable cause of MAPK activation in CRC is likely *KRAS* mutations, which account for about 40% of all CRC cases [34]. Although SW403 and HT-29 have *KRAS* or *BRAF* mutations upstream of MAPK [26], the fact that *GNA14* knockdown reduces MAPK phosphorylation in both cell lines warrants mention. Other signals can also contribute to MAPK activation independent of RAS signaling; for example, PKC can activate MAPK signaling through Raf-1 [32,35]. Since PKC is a major downstream effector of GPCRs, it is conceivable that MAPK activation by GNA14-mediated PKC activation contributes to intestinal carcinogenesis regardless of *KRAS* mutation status.

*APC* is a well-known CRC tumor suppressor gene involved in regulating β-catenin levels by forming a destruction complex with β-catenin and other factors that play key roles in the Wnt/β-catenin signaling pathway [36]. In Wnt/β-catenin signaling, it is well established that phosphorylation of β-catenin at S33, S37, and T41 and degradation via the destruction complex reduce nuclear β-catenin levels, thereby repressing the expression of β-catenin target genes [37]. However, truncated *APC* mutations prevent effective degradation of β-catenin, so accumulated nuclear β-catenin induces expression of the target genes associated with polyp formation and initiates colorectal carcinogenesis [36,38]. Indeed, human CRC cases and colorectal cancer cell lines show frequent mutations in the *APC* gene, resulting in loss-of-function [39]. The *Apc^Min/+^* heterozygote mouse has a point mutation in the *Apc* gene in which the 850th amino acid, Leu, is replaced by a stop codon and is widely used as a mouse model for CRC [40]. Interestingly, in the present study, the levels of both β-catenin and phospho-β-catenin (S675) were reduced by *GNA14* knockdown in SW403 and HT-29 cells harboring truncation mutations in *APC,* and the immunostaining of nuclear β-catenin was also attenuated in polyps of the *Gna14* knockout mouse on *Apc^Min/+^* background, indicating that GPCR signaling mediated by GNA14 may affect WNT signaling. As for the effect of GPCR signaling on WNT signaling, research has already shown that G-protein coupled lysophosphatidic acid receptors promote proliferation of colon cancer cells harboring wild-type *APC* via nuclear translocation of β-catenin through the PKC-GSK3β pathway [41]. Since phosphorylation of GSK3β at S9, which inhibits β-catenin phosphorylation and subsequent β-catenin degradation, is directly mediated by PKA and PKC [42,43], the two most common G protein effectors, GNA14 is likely to accumulate β-catenin through both pathways. Moreover, phosphorylation at S675 of β-catenin by PKA induces intranuclear accumulation of β-catenin and increases its transcriptional activity [44,45], meaning GNA14-mediated activation of PKA can modulate both the protein stabilization and transcriptional activity of β-catenin. However, in this experiment, although the amount of β-catenin protein was reduced by GNA14 knockdown, the mRNA levels of β-catenin target genes CCND1 and MYC were not reduced. This is similar to a previous report whose results state that direct knockdown of β-catenin in other colon cancer cells resulted in either a slight increase or no change in the mRNA levels of CCND1 and MYC, depending on the cell types [46], suggesting that the regulation of mRNA expression by β-catenin is complicated depending on the cellular context. Recently, a novel function of β-catenin was reported to regulate gene expression at the cap-dependent translation level [47], and whether GNA14 knockdown regulates gene expression at the translation level requires further investigation. Considering that MYC is involved in almost every aspect of the carcinogenesis process as an oncogene, the reduction of MYC protein by GNA14 knockdown is noteworthy.

Overall, a schematic diagram illustrating the effect of GNA14 on tumor progression in colon cancer is shown in Figure 7C. In the future, identifying specific GPCRs that activate GNA14 and developing antagonists for them or developing GNA14-specific modulators may be useful in the treatment of colorectal cancer.

## 5. Conclusions

In this study, we showed that knockdown or deletion of the *GNA14* gene inhibits CRC progression in cell cultures and in an in vivo mouse model. The anticancer effect of genetic inhibition of *GNA14* was valid in the context of constitutive activation of MAPK and β-catenin signaling, demonstrating the potential value of GNA14 as a therapeutic target for the treatment of colorectal cancer in the presence of *KRAS* mutations and *APC* truncating mutations.

## Data Availability

Data are contained within the article or Supplementary Materials.

## Supplementary Materials

The following supporting information can be downloaded at: https://www.mdpi.com/article/10.3390/cancers15184572/s1, Table S1. List of primers for Real-Time PCR. Table S2. List of antibodies used in western blots and dilution factors. Figure S1. Expression of GNA14 in colon cancer cell lines. (A) GNA14 mRNA levels in the indicated cells were measured by qRT-PCR analysis (n = 2). (B) The protein expression level of GNA14 was analyzed by western blotting (n = 2). Figure S2. Effect of GNA14 knockdown on cell migration. (A, B) Wound-healing assay was performed on SW403 and HT29 cells 48 h after siRNA transfection. Representative pictures taken at 0 and 24 h after removal of the block are shown. (C, D) The area was calculated at 0 and 24 h after block removal, and the relative wound healing area was expressed as a graph. Values are presented as mean ± SEM (n = 3). NS means statistically nonsignificant. Figure S3. Effect of Gna14 on cell proliferation in crypts. (A) Representative images of BrdU-stained intestinal crypt are shown (400×). Scale bar, 100 µm. (B) Each dot (right) is the average number of BrdU-positive cells per crypt in each mouse. The horizontal lines represent the averages of all mice (n = 6 per genotype). NS means statistically nonsignificant. File S1: Original western blots.
