# Supplementary material for: Tumor-Promoting Role of GNA14 in Colon Cancer Development
Source: Cancers (Basel). 2023 Sep 15;15(18):4572. doi: 10.3390/cancers15184572 (PMC10527020; doi:10.3390/cancers15184572)

Table S1. List of primers for Real-Time PCR.

Target genes	Sense (5' to 3')	Antisense (5' to 3')
CCND1	AATCCGCCCTCCATGGTG	CCAGCAGGGCTTCGATCTG
GNA14	AGGGAGTACCAGCTGTCGGA	TTGGGTAGGCACGAATGA
Gna14 (mouse)	AAGGGCTTCACGAAGCTGGT	GGGCATTTTCCTTATTCTGC
MYC	CTTCTCTCCGTCCTCGGATTCT	GAAGGTGATCCAGACTCTGACCTT
POLR2A	GCACCACGTCCAATGACAT	GTGCGGCTGCTTCCATAA
RNA18SN1	TCAACTTTCGATGGTAGTCGCC	GGCCTCGAAAGAGTCCTGTATTGT

POLR2A was used as an endogenous control for human genes, and *RNA18SN1* was used as an endogenous control for mouse gene.

Table S2. List of antibodies used in western blots and dilution factors.

Target Antibodies	Company	Cat No.	Dilution factor
AKT	Cell Signal Technology	4691	1:2,000
phospho-AKT (T308)	Cell Signal Technology	9275	1: 2,000
phospho-AKT (S473)	Cell Signal Technology	9271	1: 1,000
β-catenin	Cell Signal Technology	9582	1: 20,000
phospho-β-catenin (S675)	Cell Signal Technology	4176	1: 50,000
CCND1	Invitrogen	MA5-14512	1: 2,000
ERK1/2	Cell Signal Technology	9102	1: 2,000
phosphor-ERK1/2 (T202/Y204)	Cell Signal Technology	9101	1: 20,000
GAPDH	AB Frontier	LF-PA0018	1: 6,000
GNA14	MyBioSource	MBS7005189	1: 5,000
MYC	Santa Cruz	sc-40	1:2,000

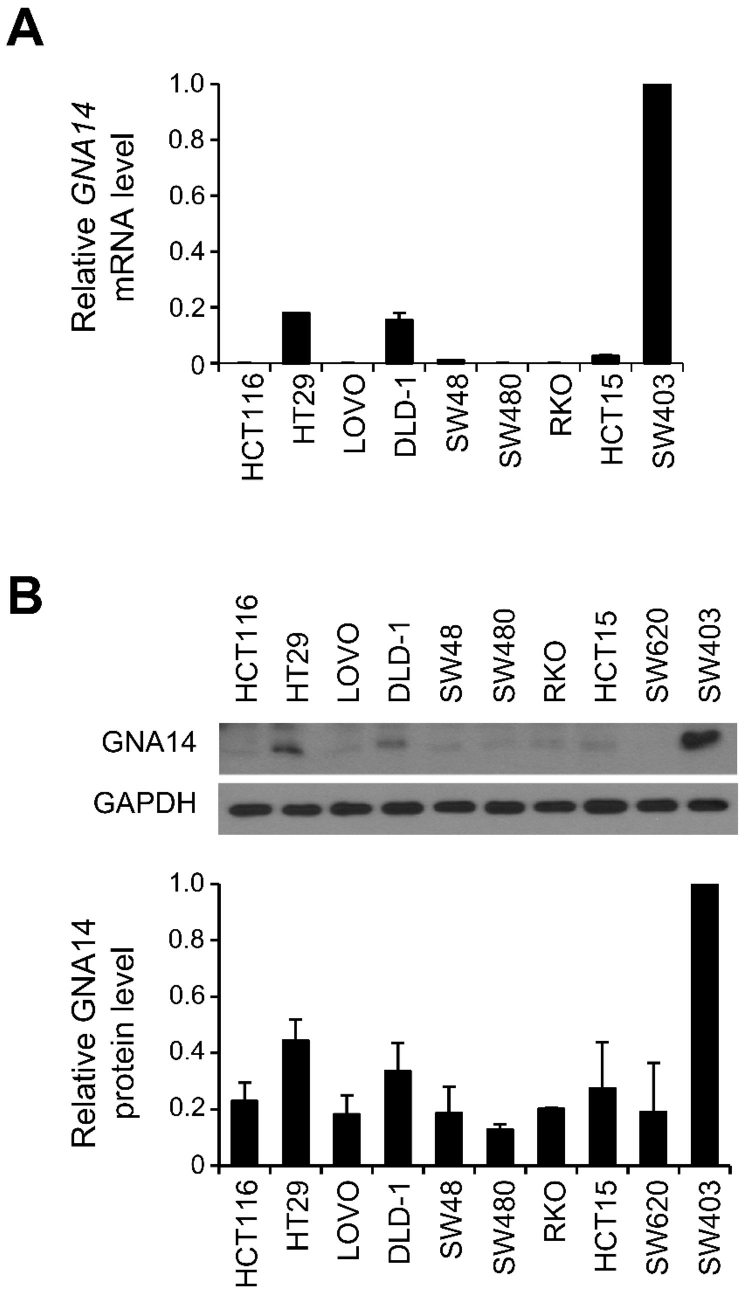

Figure S1. Expression of *GNA14* in colon cancer cell lines. (A) *GNA14* mRNA levels in the indicated cells were measured by qRT-PCR analysis (n = 2). (B) The protein expression level of *GNA14* was analyzed by western blotting (n = 2).

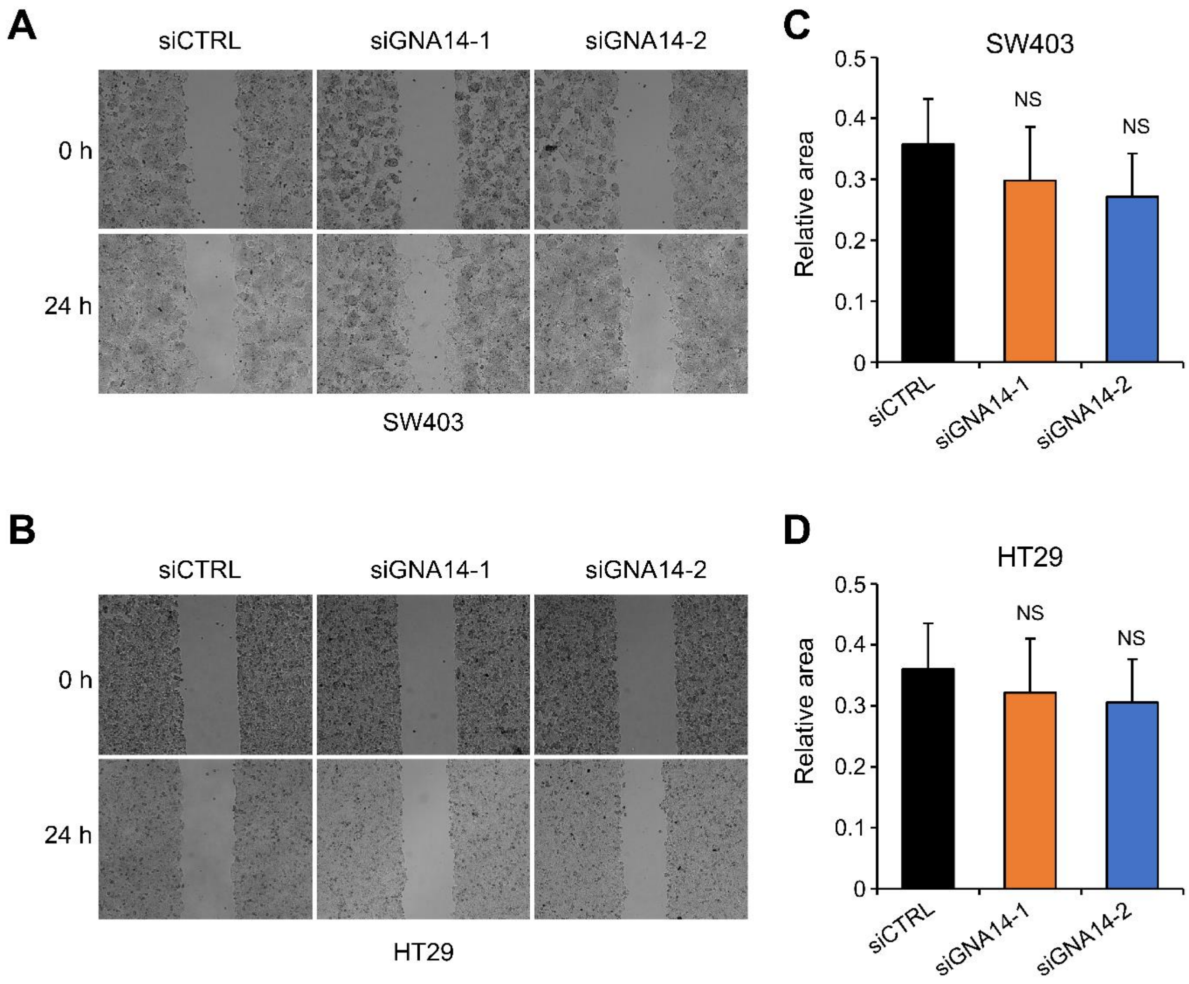

Figure S2. Effect of *GNA14* knockdown on cell migration. (A, B) Wound-healing assay was performed on SW403 and HT29 cells 48 h after siRNA transfection. Representative pictures taken at 0 and 24 h after removal of the block are shown. (C, D) The area was calculated at 0 and 24 h after block removal, and the relative wound healing area was expressed as a graph. Values are presented as mean ± SEM (n = 3). NS means statistically nonsignificant.

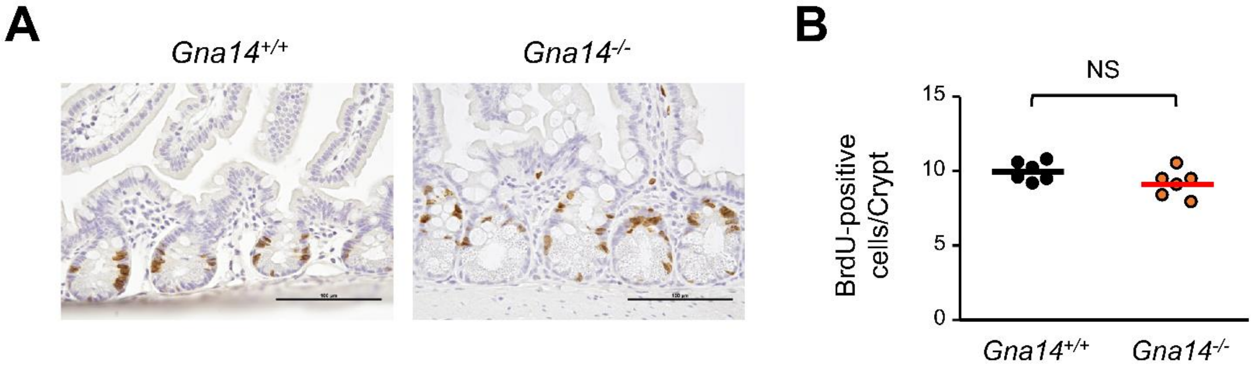

Figure S3. Effect of *Gna14* on cell proliferation in crypts. (A) Representative images of BrdU-stained intestinal crypt are shown (400x). Scale bar, 100 µm. (B) Each dot (right) is the average number of BrdU-positive cells per crypt in each mouse. The horizontal lines represent the averages of all mice (n = 6 per genotype). NS means statistically nonsignificant.

File S1: Original western blots.

Original pictures

Figure 1B

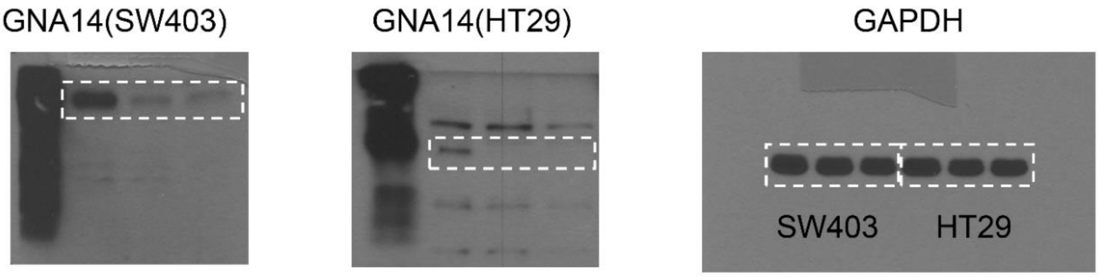

Figure S1B

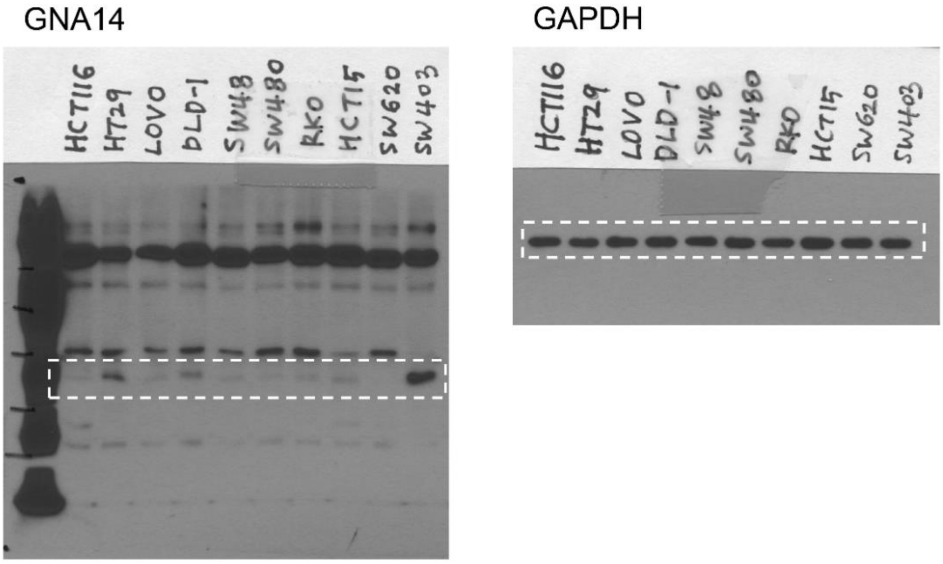

Figure 2B

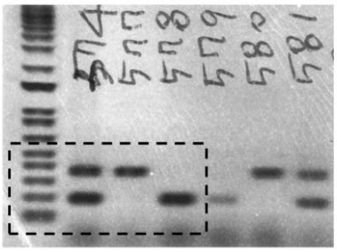

Figure 2D

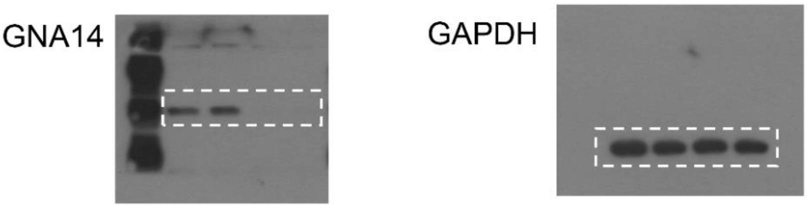

Figure 6A

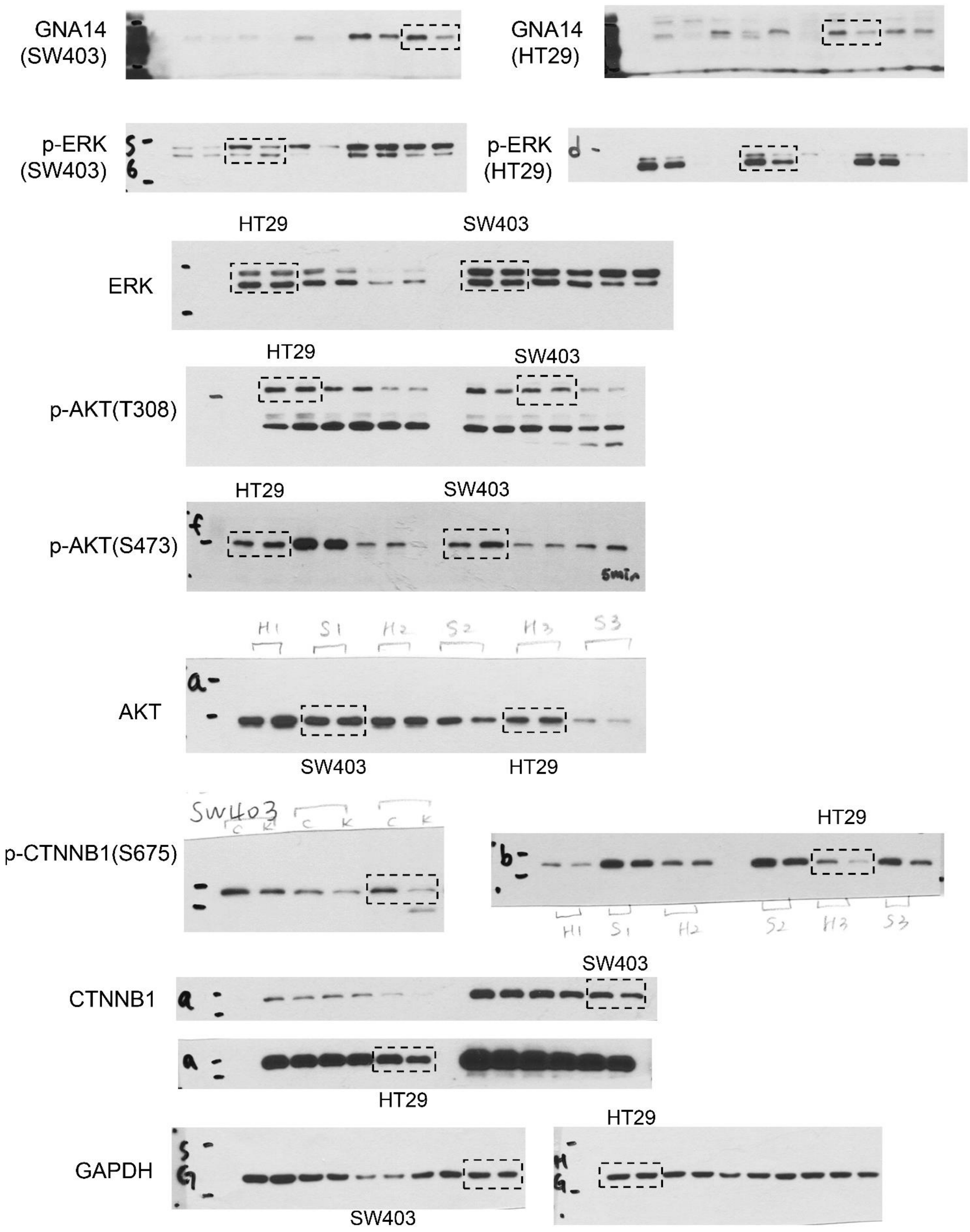
GNA14 (SW403)
GNA14 (HT29)
p-ERK (SW403)
p-ERK (HT29)
HT29
SW403
ERK
HT29
SW403
p-AKT(T308)
HT29
SW403
p-AKT(S473)
AKT
SW403
HT29
p-CTNNB1(S675)
HT29
CTNNB1
SW403
HT29
GAPDH
HT29
SW403

Figure 6D

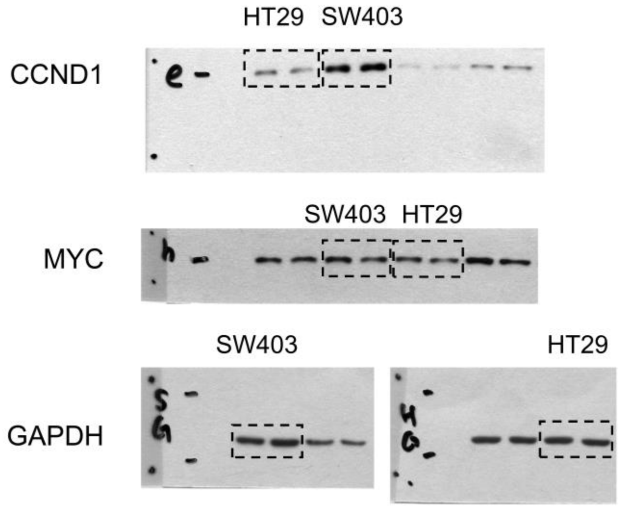

Supplement: Supplementary file 1 [file cancers-15-04572-s001.zip › cancers-2595456-SI.pdf]
